# Natural Products Modulate Cell Apoptosis: A Promising Way for the Treatment of Ulcerative Colitis

**DOI:** 10.3389/fphar.2022.806148

**Published:** 2022-01-31

**Authors:** Chenhao Liu, Yiwei Zeng, Yulong Wen, Xinggui Huang, Yi Liu

**Affiliations:** ^1^ School of Basic Medical Sciences, Chengdu University of Traditional Chinese Medicine, Chengdu, China; ^2^ School of Acupuncture-Moxibustion and Tuina, Chengdu University of Traditional Chinese Medicine, Chengdu, China

**Keywords:** ulcerative colitis, inflammatory bowel disease, apoptosis, natural products, intestinal epithelia cells

## Abstract

Ulcerative colitis (UC) is a chronic inflammatory bowel disease impacting patients’ quality of life and imposing heavy societal and economic burdens. Apoptosis of intestinal epithelial cells (IECs) has been considered an early event during the onset of UC and plays a crucial role in disease development. Thus, effectively inhibiting apoptosis of IECs is of critical significance for the clinical management of UC, presenting a potential direction for the research and development of pharmacotherapeutic agents. In recent years, research on the ameliorative effects of natural products on UC through inhibiting IECs apoptosis has attracted increasing attention and made remarkable achievements in ameliorating UC. In this review, we summarized the currently available research about the anti-apoptotic effects of natural products on UC and its mechanisms involving the death-receptor mediated pathway, mitochondrial-dependent pathway, ERS-mediated pathway, MAPK-mediated pathway, NF-κB mediated pathway, P13k/Akt pathway, JAK/STAT3 pathway, and NLRP3/ASC/Caspase-1 pathway. Hopefully, this review may yield useful information about the anti-apoptotic effects of natural products on UC and their potential molecular mechanisms and provide helpful insights for further investigations.

## 1 Introduction

Ulcerative colitis (UC) is a chronic inflammatory bowel disease characterized by relapsing and remitting mucosal inflammation restricted to the colon and rectum. It is a global health challenge, and its typical clinical presentation consists of diarrhea, hematochezia, and abdominal pain with histological features of diffuse inflammation limited to mucosa and submucosa, crypt abscesses, crypt architectural distortion, mucin depletion, and goblet cell depletion (Conrad et al., 2014; Yu and Rodriguez, 2017). The prevalence of UC varies in different regions. A systematic review of population-based studies demonstrated that the prevalence ranges from 1.2–57.3 per 100,000 in Asia to14.5–505.0 per 100,000 and 139.8–286.3 per 100,000 in Europe and North America, respectively (Ng et al., 2017). UC can occur at any age and is most commonly diagnosed in the second to fourth decade of life (Du and Ha, 2020). Although the specific etiopathogenesis of UC remains obscure, it has been well-recognized that a complex interaction of intestinal microbiota, genetic susceptibility, and environmental factors may disturb the immune system and result in the immune-mediated chronic intestinal inflammatory response (Adams and Bornemann, 2013; Ananthakrishnan, 2015). The long-term inflammation leads to irreversible bowel damage and a higher risk of poor outcomes, such as colectomy and colorectal cancer (Rioux, 2008; Bopanna et al., 2017), impacting patients’ quality of life, imposing heavy social and economic burdens, even increasing mortality. Conventional treatments for UC, including aminosalicylates, corticosteroids, and immunosuppressants, only induce and maintain remission and often elicit adverse effects (Wan et al., 2014; Burri et al., 2020). Several bioagents have been developed to target such molecular mechanisms as tumor necrosis factor (TNF), integrin, and Janus kinase (JAK) (Pugliese et al., 2017; Wehkamp and Stange, 2018). However, these therapeutic agents have primarily focused on the inflammatory cascades to alleviate the disease process rather than histological healing, and most of these substances are expensive for the medium- and low-income populations. Therefore, researching effective and affordable medications with fewer side effects for UA is urgently needed.

Previous studies have demonstrated that the apoptosis of intestinal epithelial cells (IECs) in the colon contributes to chronic inflammatory bowel diseases. IECs play a significant role in host defense, mucosal homeostasis maintenance, and immune response (Eissa et al., 2019; Zhang J. et al., 2020). The intestinal mucosa structure is maintained by a sensitive balance between the apoptosis and proliferation of epithelial cells, which may be disturbed in the inflammatory intestine due to the increased proinflammatory cytokines, including tumor necrosis factor (TNF), interleukin (IL), and interferon family members (Schulzke et al., 2006; Qiu et al., 2011). Apoptosis of IECs has been considered an early event during the onset of UC and plays a crucial role in disease development (Iwamoto et al., 1996). Both extrinsic and intrinsic apoptotic pathways are involved in the UC pathology and regulated by multiple signaling pathways. IECs act as a barrier between lumina and the external environment. Excessive epithelial apoptosis disrupts the epithelial defense system and may cause the breakdown of epithelial barrier function, which may facilitate the mucosal invasion of intraluminal microorganisms and luminal antigen uptake (Hagiwara et al., 2002; Schulzke et al., 2009; Seidelin and Nielsen, 2009; Araki et al., 2010) and potentiate the prolonged inflammatory response. Therefore, effectively inhibiting apoptosis of IECs and retaining the integrated epithelial barrier are of critical significance for the clinical management of UC, presenting a potential direction for the research and development of pharmacotherapeutic agents (Verstege et al., 2006).

Recently, natural products, including extracts and isolated metabolites from medicinal botanical drugs and plants, have drawn increasing attention for their potential therapeutic effects on UC with high availability and fewer side effects (Wan et al., 2014; Santana et al., 2017). These natural agents have been demonstrated to possess anti-inflammatory, antioxidative, anti-apoptotic, antiplatelet, and immune-regulatory properties through mediating multiple signaling pathways related to the pathogenesis of UC (Ke et al., 2012; Triantafyllidi et al., 2015; Cao et al., 2019). Furthermore, several extracts and isolated metabolites from natural products have been reported to exert amelioratory effects on UC through inhibiting IECs apoptosis with multiple pathways, including death receptor-mediated pathway, mitochondria-dependent pathway, endoplasmic reticulum stress-mediated pathway, MAPK-mediated pathway, NF-κB mediated pathway, and P13K/Akt mediated pathway. Therefore, to yield helpful insights for further research and development of novel and efficacious pharmaceutic intervention in this field, a comprehensive review of the anti-apoptotic activities of natural products on UC and their potential molecular mechanisms is necessary. In this review, the following electronic databases were searched from the inception to July 2021 to identify the eligible studies: PubMed, Embase, Web of Science, China National Knowledge Infrastructure, China Biomedical Literature Database, Wanfang Database, VIP database, and Chinese Scientific Journals Database. The following terms were used in a combination for the search: Colitis, Ulcerative, ulcerative colitis, inflammatory bowel disease, colitis, apoptosis, apoptotic, cell death, natural product, natural medicine, traditional medicine, and traditional Chinese medicine. A meticulous review was performed, and the quality of all the included studies was assessed in accordance with the Best practice in research—Overcoming common challenges in phytopharmacological research (Heinrich et al., 2020). The detailed information of natural products and their potential effects with mechanisms on modulating apoptosis in UC is illustrated in Tables 1, 2, and the chemical structures of isolated metabolites are summarized in Table 3.

**TABLE 1:** Anti-apoptotic activities of natural products on UC-induced intestinal epithelial apoptosis.

Potential mechanisms	Detailed mechanisms	Extracts/Isolated metabolites (dose/concentration)	Cells/Animal	Related targets	Refs
Death receptor apoptotic pathway	Down-regulating Fas and FasL; Down-regulating caspase-3, -9, and bax; Up-regulating bcl-2	Baicalin (30–120 mg/kg)	RAW264.7 cells stimulated by lipopolysaccharide; 2,4,6-trinitrobenzene sulfonic acid-induced rats	Fas, FasL, caspase-3, -9, bax, bcl-2	Yao et al. (2016)
	Down-regulating Fas; Increasing FasL, bcl-2	Sishen Wan (2.5, 5, 10 g/kg)	Sprague-Dawley rats stimulated by TNBS	Fas, FasL, bcl-2	Liu et al. (2011)
	Down-regulating Fas, FasL, bax, caspase-3; up-regulating bcl-2	Iridoid Glycosides (80, 160 and 240 mg/kg)	Sprague-Dawley rats stimulated by DSS	Fas, FasL, caspase-3, bcl-2	Liu and Wang, (2011)
	Decreasing Fas, FasL, caspase-3, bax; Increasing bcl-2	Wumei Pill (13.3–53.2 g/kg)	Sprague-Dawley rats stimulated by TNBS	Fas, FasL, caspase-3, bax, bcl-2	(Shuguang et al., 2016; Yi et al., 2016)
Mitochondria-dependent apoptotic pathway	Down-regulating bax; Up-regulating bcl-2	Aucklandia and Coptis Pills (1.6 g/kg)	Wistar rats stimulated by TNBS	Bax, bcl-2	Yan and Jingen., (2016)
	Down-regulating bax, caspase-3	Hesperetin (100 mg/kg)	Wistar rats stimulated by TNBS	Bax, caspase-3	Polat and Karaboga, (2019)
	Down-regulating cyt-c, caspase-9, -3, bcl-2/bax	Laggera Alata Flavone (100, 200, 400 mg/kg)	Sprague Dawley rats stimulated by TNBS	cyt-c, caspase-9, -3, bcl-2, bax	Xiaobin and Xiaodong., (2014)
	Down-regulating bax; Up-regulating bcl-2	Astragalus Polysaccharide (200 mg/kg)	Wistar rats stimulated by TNBS	Bax, bcl-2	Weijie et al. (2019)
	Down-regulating bax, caspase-3; Up-regulating bcl-2	Indigo (200, 400, 800 mg/kg)	C57BL/6 mice stimulated by DSS	Bax, caspase-3, bcl2	Wenqiang et al. (2019)
	Decreasing bax mRNA expression; Increasing bcl-2 mRNA expression	Aloe Vera Gel (200 mg/kg)	Sprague Dawley rats stimulated by 3% acetic acid	Bax, bcl-2	Hassanshahi et al. (2020)
	Decreasing bax, caspase-3	Coptidis Rhizoma and Magnoliae Officinalis Cortex (1, 2, 4 g/kg)	Sprague Dawley rats stimulated by TNBS	Bax, caspase-3	Xian-juan et al. (2020)
	Up-regulating bcl-2; Down regulating bax and caspase-3	Qingchang Wenzhong granule (0.42–2.20 g/kg)	Male Sprague Dawley rats stimulated by DSS	Bcl-2, bax, caspase-3	Shi et al. (2019)
	Down-regulating bax, caspase-3; Up-regulating bcl-2	Artesunate (30 mg/kg)	Female ICR mice stimulated by DSS	Bcl-2, bax, caspase-3	Yin et al. (2020)
	Decreasing bax, caspase-3; Increasing bcl-2, bcl-xL	Plumericin (0.5–2μg; 3 mg/kg)	IEC-6 cells induced by LPS and IFN; Male CD1 mice stimulated by DNBS	Bcl-2, bax, caspase-3	Rapa et al. (2021)
	Decreasing caspase-3, bax; Increasing bcl,2	Graviola (100 mg/kg)	Male Wistar rats induced by acetic acid	caspase-3, bax, bcl,2	Helal and Abd Elhameed, (2021)
Endoplasmic reticulum stress-mediated pathway	Down-regulating GRP78, caspase-3, -12	Berberine (100, 150, 200 mg/kg; 10 ml/kg)	Male BALB/c mice stimulated by DSS	GRP78, caspase-3, -12	(Yan et al., 2018; Yan et al., 2020)
	Decreasing GRP78, caspase-3, -12	Glycyrrhizin (0.5, 1, 2 mmol/L)	IECs induced by H2O2; Male BALB/c mice stimulated by DSS	GRP78, caspase-3, -12	Yan and Bin., (2020)
	Decreasing GRP78, PERK, CHOP, caspase-3, -12	Ginsenoside Rb1 (20,40 mg/kg)	C57BL/6 mice stimulated by DSS; IEC-6 rat intestinal epithelial cells induced by TNBS	GRP78, PERK, CHOP, caspase-3, -12	Dong et al. (2021)
	Inhibiting PERK-ATF4-CHOP pathway	Limonin (25, 50, 100 mg/kg)	Female C57BL/6 mice stimulated by DSS; RAW 264.7 cells induced by LPS	p-PERK, *p*-eIF2α, ATF4, CHOP	Song et al. (2021)
	Decreasing p-PERK, *p*-eIF2α, ATF4, CHOP, bax	Gancao Xiexin Decoction (10, 20,40 μL)	Caco-2 cells Male BALB/c mice stimulated by DSS	PERK, eIF2α, ATF4, CHOP, bax	Yan et al. (2021)
	Decreasing GRP78, CHOP, PERK, eIF2α, ATF4, XBP1s, capsase-12	Artesuante (30 mg/kg)	Female ICR mice stimulated by DSS	GRP78, CHOP, PERK, eIF2α, ATF4, XBP1s, capsase-12	Yin et al. (2021)
MAPK-mediated pathway	Suppressing p38, ERK1/2, and MAP2K1	SNE (50, 200 mg/kg)	Male ICR mice stimulated by DSS	p38, ERK1/2, MAP2K1	Taya et al. (2016)
	Modulating p38-, JNK-MAPK pathways	Curcumin (100 mg/kg)	Male Wistar albino rats stimulated by acetic acid	p38, JNK	Topcu-Tarladacalisir et al. (2013)
	Decreasing p38, p53, c-jun, c-fos, bax, caspase-3; Increasing bcl-2	Si ShenWan (5 g/kg)	C57/BL mice stimulated by TNBS	p38, c-jun, c-fos, bax, caspase-3, bcl-2	Zhao et al. (2013)
	Suppressing p38; down-regulating caspase-3; up-regulating PPARγ	Geraniol (250 mg/kg)	Male Wistar rats stimulated by TNBS	p38, caspase-3, PPARγ	Soubh et al. (2015)
	Inhibiting MAPK/NF-κB pathway; Up-regulating bcl-2; Down regulating bax and caspase-3, -9	Paeoniflorin (15, 30, 45 mg/kg; 2.5 g/kg)	Male Balb/c mice stimulated by TNBS; Male Wistar rats stimulated by DSS	ERK, p38, blc-2, bax, caspase-3, -9	(Gu et al., 2017; (Lanzhen et al., 2020)
	Inhibiting MAPK/NF-κB pathway; Increasing ERK1/2, *p*-ERK, p38, p-p38, JNK, *p*-JNK, *p*-IκB, p-p65 Decreasing cleaved caspase-3; Increasing bcl-2	Indirubin (10 mg/kg); Isatin (10 mg/kg)	Male BALB/c mice stimulated by DSS	ERK, p38, JNK, caspase-3, bcl-2	Gao et al. (2018)
	Increasing ERK1/2, p-ERK, p38, p-p38, JNK, p-JNK, p-I𝜅B, p-p65	Chlorogenic Acid (30, 60, 120 mg/kg)	C57BL/6 mice stimulated by DSS	ERK, p38, JNK, p65	Gao et al. (2019)
	Inhibiting *p*-JNK, p-p38; Increasing bcl-2; Decreasing bax	Berberis lycium fruit extract (125–500 mg/kg)	Balb/C mice stimulated by DSS	JNK, p38, bcl-2, bax	Sharma et al. (2020)
	Inhibiting S100A9/MAPK/NF-κB pathway; Increasing bcl-2; Decreasing bax, caspase-3, p53	Anemoside B4 (5, 10 mg/kg)	SD rats stimulated by TNBS	S100A9, TLR4, JNK, p65, blc-2, bax, caspase-3, p53	(Yong et al., 2020; Zhang et al., 2021)
NF-κB mediated pathway	Inhibiting IκBα degradation, caspase-3 activation	Deoxyschisandrin (1–5 μg/ml)	HCT116 cells induced by H2O2	IκBα, caspase-3	Gu et al. (2010)
	Inhibiting IκBα degradation; Down-regulating caspase-3, -9	Corilagin (7.5, 15, 30 mg/kg)	Male C57BL/6 mice stimulated by DSS	IκBα, caspase-3, -9	Xiao et al. (2013)
	Inhibiting IκBα, and IKKβ Down-regulating Fas/FasL, bax, caspase-3; Up-regulating bcl-2	Iridoid Glycosides Fraction (80, 160 and 240mg/kg)	Sprague-Dawley rats stimulated by DSS	IκBα, IKKβ, Fas, FasL, Bax, caspase-3, bcl-2	(Liu and Wang, 2011; Zhang et al., 2020a)
	Down-regulating NF-kBp65, bax, caspase-3; Up-regulating bcl-2	Portulaca Extract (100 mg/kg)	Female mice stimulated by DSS	NF-kBp65, bax, bcl-2, caspase-3	Kong et al. (2018)
	Decreasing p-p65, caspase-3	QingBai decoction (0.0195 ml/g)	C57/bL mice stimulated by DSS	NF-kBp65, caspase-3	Lin et al. (2019)
	Inhibiting *p*-IκBα, p-p65; Decreasing caspase-3, -9; Increasing bcl-2	Gallic acid (20, 40, 60 mg/kg, mg/ml)	Balb/c mice stimulated by TNBS HIEC-6 cells induced by IL-1β	IκBα, NF-kBp65, caspase-3, -9, bcl-2	Zhu et al. (2019a)
	Suppressing NF-kB phosphorylation; Decreasing PARP	C. arietinum ethanol Extract (100, 200 mg/kg)	Male ICR mice stimulated by DSS	NF-kBp65, PARP	Kim et al. (2020)
	Down-regulating NF-kB, bax; Up-regulating bcl-2	Oleuropein (350 mg/kg)	Male laboratory albino rats stimulated by acetic acid	NF-kB, bax, bcl-2	Motawea et al. (2020)
	Suppressing NF-κBp65, pNF-κB, ERK1/2, COX-2 Down-regulating caspase-3	6,7-Dihydroxy-2,4-Dimethoxyphenanthrene (60,120, 240 mg/kg)	Male BALB/c mice stimulated by DSS	NF-κBp65, pNF-κB, ERK1/2, COX-2, caspase-3	Li et al. (2021)
	Down-regulating TLR4, NF-κB, caspase-3; Suppressing NLPR3, cleaved caspase-1, ASC mRNA	Canna x generalis L.H. Bailey rhizome extract (100, 200 mg/kg)	Mice stimulated by DSS	TLR4, NF-κB, NLPR3, ASC mRNA, caspase-3, -1	Mahmoud et al. (2021)
	Inhibiting NF-κBp65, IκBα Down-regulating bax, caspase-3, cyto-c; Up-regulating bcl-2	Coptisine (100 mg/kg)	Male BALB/c mice stimulated by DSS	NF-κBp65, IκBα, ax, caspase-3, cyto-c, bcl-2	Wang et al. (2021)
	Decreasing NF-κBp65, *p*-IKKβ/IKKβ, *p*-IKBα/IKBα; Decreasing cyt-c, caspase-3, -9, bcl-2/bax	Baicalin (30, 60, 90 mg/kg)	RAW264.7 cells induced by LPS; Sprague Dawley rats stimulated by TNBS	NF-κBp65, IKBα, IKKβ, cyt-c, caspase-3, -9, bcl-2, bax	Shen et al. (2019)
	Inhibiting TLR4, NF-κB; Decreasing bax, caspase-3; Increasing bcl-2	Deoxyschizandrin (20, 40, 80 mg/kg)	Sprague Dawley rats stimulated by DSS	TLR4, NF-κB, bcl-2, bax, caspase-3	Yu and Qian, (2021)
	Decreasing NF-κBp65, caspase-3	Hyperoside (25, 50, 100 mg/kg)	Wistar rats stimulated by TNBS	NF-κBp65, caspase-3	Yu et al. (2021)
P13K/Akt pathway	Regulating P13K/Akt pathway; Decreasing caspase-9, FasL	Baicalin (20, 50, 100 mg/kg)	Male Sprague Dawley rats stimulated by TNBS	P13K, Akt, caspase-9, FasL	Zhu et al. (2017)
	Regulating PI3K/Akt activation	Oxymatrine (25, 50, 100 mg/kg)	Male BALB/c mice stimulated by DSS	PI3K, Akt	Chen et al. (2017)
	Promoting P13K, Akt activity Down-regulating caspase-3, bad; Up-regulating bcl-2, p53	Costus root granules (1,000 mg/kg)	Male Sprague Dawley rats stimulated by DSS	PI3K, Akt, caspase-3, bcl-2, bax, p53	Wang et al. (2018)
	Regulating Akt; Decreasing caspase-3, -9, PARP	Luteolin (50, 100 mg/kg)	Male C57BL/6 mice stimulated by DSS	Akt, caspase-3, -9, PARP	Vukelic et al. (2020)
Other	Decreasing caspase-3, -8	polysaccharide of Portulaca oleracea (200mg/0.33 ml)	Male Sprague Dawley rats stimulated by TNBS	Caspase-3, -8	Feng et al. (2010)
	Decreasing caspase-3	Honey (5 g/kg)	Male albino Wistar rats stimulated by DSS	Caspase-3	Nooh and Nour-Eldien, (2016)
	Inhibiting JAK2/STAT3 pathway	Aloe polysaccharide (15 mg/kg)	HT-29 cell induced by LPS; Male SD rats stimulated by TNBS	JAK2, *p*-JAK2, STAT3, p-STAT3	Lin et al. (2017)
	Up-regulating Sonic hedgehog signaling pathway; Decreasing caspase-3, bax; Increasing bcl-2	Polydatin (15, 30, 45 mg/kg)	Male C57BL/6 mice stimulated by DSS	Shh, caspase-3, bcl-2, bax	Lv et al. (2018)
	Down-regulating *p*-JAK2, pSTAT3, caspase-3, -9; Up-regulating bcl-2, bcl-xL	Tripterygium glycosides (27 mg/kg)	Male Sprague Dawley rats stimulated by TNBS	JAK2, STAT3, caspase-3, -9, bcl-2, bcl-xL	Nan et al. (2019)
	Down-regulating bax; Up-regulating bcl-2	Hydroxytyrosol (50 mg/kg)	Male laboratory albino rats stimulated by acetic acid	Bcl-2, bax	Elmaksoud et al. (2021)
	Inhibiting IL-10/JAK1/STAT3 pathway	Chushi Jianpi decoction (1 ml/kg)	BALB/c mice stimulated by DSS	IL-10, JAK, STAT3	Chen et al. (2021)
	Decreasing bax, caspase-3, TLR4, MyD88; Increasing bcl-2	Crocin (0.05, 0.1 g/kg)	Male Sprague Dawley rats stimulated by DSS	Bax, caspase-3, TLR4, MyD88, bcl-2	Yang et al. (2020)
	Decreasing bax, caspase-3, -9; Increasing VLDLR, bcl-2	Tanshinol (15, 30 mg/kg)	Male C57BL/6J mice stimulated by DSS	VLDLR, bax, bcl-2, caspase-3, -9	Zhu et al. (2021)
	Decreasing NRLP3, ASC, caspase-1	Walnut oil (2.5 ml/kg)	Kunming male mice stimulated by DSS	NRLP3, ASC, caspase-1	Miao et al. (2021)

TNBS, trinitrobenzene sulfonic acid; PPARγ, peroxisome proliferator activated receptor; CHOP, C/EBP, homologous protein; GRP78, glucose-regulated protein 78; PERK, protein kinase R-like ER, kinase; ATF4, activating transcription factor; JNK, c-jun N-terminal kinase; DSS, dextran sulfate sodium; DNBS, dinitrobenzenesulfonate; SNE, spirogyra neglecta extract; TLR4, toll-like receptor 4; IL-8, interleukin-8; PPAR-γ, peroxisome proliferator-activated receptor-γ; Cyto-c, cytochrome-c; LPS, lipopolysaccharide; TRAF6, tumor necrosis factor receptor-associated factor 6; NLRP3, NOD-like receptor protein; ASC, apoptosis-associated speck-like protein containing CARD; RJ, rumex japonicus houtt; JAK, janus kinase; STAT3, signal transducer and activator of transcription 3; IFN, interferon-γ; VLDLR, very low density lipoprotein receptor; Hh, Hedgehog.

**TABLE 2:** Components of TCM prescriptions.

Prescription	Components [dosage(g)/Concentrations (%)]	Scientific name	Refs
Sishen Wan	Wu Zhu Yu (6.67%)	Rutaceae: *Tetradium ruticarpum (A.Juss.) T.G.Hartley*	Liu et al. (2011), Zhao et al. (2013)
	Bu Gu Zhi (26.67%)	Fabaceae: *Cullen corylifolium (L.) Medik*	
	Wu Wei Zi (13.33%)	Schisandraceae: *Schisandra chinensis (Turcz.) Baill*	
	Rou Dou Kou (13.33%)	Myristicaceae: *Myristica fragrans Houtt*	
	Sheng Jiang (26.67%)	Zingiberaceae: *Zingiber officinale Roscoe*	
	Da Zao (13.33%)	Rhamnaceae: *Ziziphus Jujuba Mill*	
Wumei Pill	Wu Mei (16 g)	Rosaceae: *Prunus mume (Siebold) Siebold and Zucc*	Yi et al. (2016)
	Xi Xin (6 g)	Aristolochiaceae: *Asarum heterotropoides F.Schmidt*	
	Gan Jiang (10 g)	Zingiberaceae: *Zingiber officinale Roscoe*	
	Gui Zhi (6 g)	Lauraceae: *Neolitsea cassia (L.) Kosterm*	
	Fu Zi (6 g)	Ranunculaceae: *Aconitum carmichaeli Debeaux*	
	Shu Jiao (4 g)	Rutaceae: *Zanthoxylum bungeanum Maxim*	
	Huang Lian (16 g)	Ranunculaceae: *Coptis chinensis Franch*	
	Ren Shen (6 g)	Araliaceae: *Panax ginseng C.A.Mey*	
	Dang Gui (4 g)	Apiaceae: *Angelica sinensis (Oliv.) Diels*	
	Huang Bo (6 g)	Rutaceae: *Phellodendron amurense Rupr*	
Qingchang Wenzhong granule	Huang Lian (6 g)	Ranunculaceae: *Coptis chinensis Franch*	Shi et al. (2019)
	Pao Jiang (10 g)	Zingiberaceae: *Zingiber officinale Roscoe*	
	Ku Shen (15 g)	Fabaceae: *Sophora flavescens Aiton*	
	Qing Dai (6 g)	Brassicaceae: *Isatis tinctoria subsp. tinctoria*	
	Di Yu (15 g)	Rosaceae: *Sanguisorba officinalis L*	
	Mu Xiang (6 g)	Asteraceae: *Dolomiaea costus (Falc.) Kasana and A.K.Pandey*	
	San Qi (6 g)	Araliaceae: *Panax notoginseng (Burkill) F.H.Chen*	
	Gan Cao (6 g)	Fabaceae: *Glycyrrhiza glabra L*	
Gancao Xiexin decoction	Gan Cao (12 g)	Fabaceae: *Glycyrrhiza glabra L*	Yan et al. (2021)
	Gan Jiang (9 g)	Zingiberaceae: *Zingiber officinale Roscoe*	
	Ban Xia (9 g)	Araceae: Pinellia ternata (Thunb.) Makino	
	Huang Qin (9 g)	Lamiaceae: *Scutellaria baicalensis Georgi*	
	Huang Lian (3 g)	Ranunculaceae: *Coptis chinensis Franch*	
	Dang Shen (9 g)	Lamiaceae: *Salvia miltiorrhiza Bunge*	
	Da Zao (6 g)	Rhamnaceae: *Ziziphus Jujuba Mill*	
Qingbai decoction	Da Qing Ye (12 g)	Brassicaceae: *Isatis tinctoria subsp. Tinctoria,* leaves	Lin et al. (2019)
	Ban Lan Gen (20 g)	Brassicaceae: *Isatis tinctoria subsp. Tinctoria,* roots	
	Huang Bo (9 g)	Rutaceae: *Phellodendron amurense Rupr*	
	Ku Shen (20 g)	Fabaceae: *Sophora flavescens Aiton*	
	Yi Ren (30 g)	Poaceae: *Coix lacryma-jobi L*	
	Wu Zei Gu (25 g)	Cuttlebone	
Chushi Jianpi decoction	Bai Zhu (5 g)	Asteraceae: *Atractylodes macrocephala Koidz*	Chen et al. (2021)
	Cang Zhu (3 g)	Asteraceae: *Atractylodes lancea (Thunb.) DC.*	
	Fu Ling (3 g)	Smilacaceae: *Smilax glabra Roxb*	
	Bai Shao (3 g)	Paeoniaceae: *Paeonia lactiflora Pall*	
	Dang Gui (2 g)	Apiaceae: *Angelica sinensis (Oliv.) Diels*	
	Hou Po (2 g)	Magnoliaceae: *Magnolia officinalis Rehder and E.H.Wilson*	
	Chen Pi (2 g)	Rutaceae: *Citrus x aurantium L*	
	Zhu Ling (1.5 g)	Pteridaceae: *Adiantum capillus-veneris L*	
	Ze Xie (1.5 g)	Alismataceae: *Alisma plantago-aquatica L*	
	Chai Hu (2 g)	Apiaceae: *Bupleurum chinense DC.*	
	Sheng Ma (2 g)	Ranunculaceae: *Actaea cimicifuga L*	
	Fang Feng (2 g)	Apiaceae: *Saposhnikovia divaricata (Turcz. ex Ledeb.) Schischk*	
	Gan Cao (1 g)	Fabaceae: *Glycyrrhiza glabra L*	

**TABLE 3:** Chemical structures of natural products.

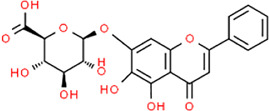 Baicalin	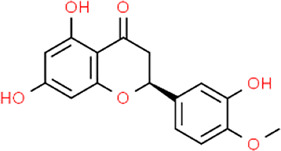 Hesperetin	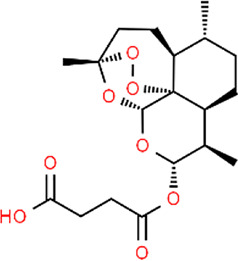 Artesunate	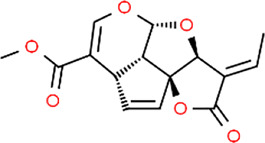 Plumericin
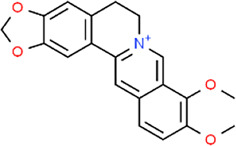 Berberine	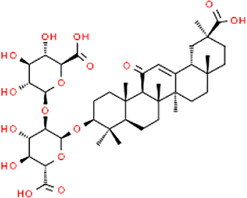 Glycyrrhizin	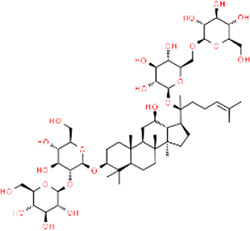 Ginsenoside Rb1	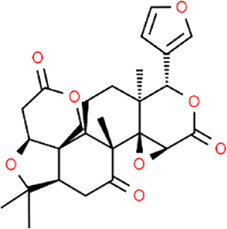 Limonin
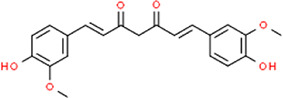 Curcumin	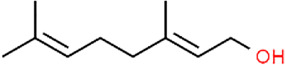 Geraniol	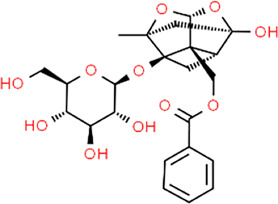 Paeoniflorin	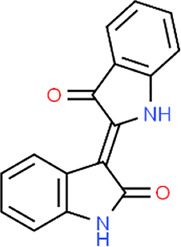 Indirubin
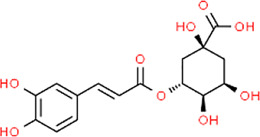 Chlorogenic Acid	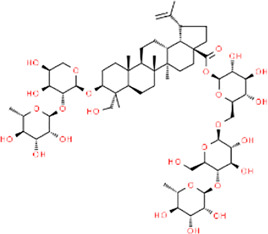 Anemoside B4	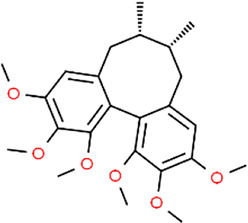 Deoxyschisandrin	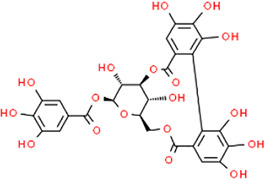 Corilagin
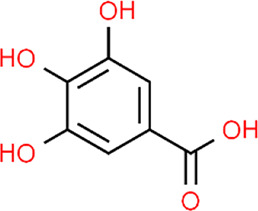 Gallic acid	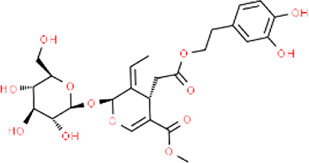 Oleuropein	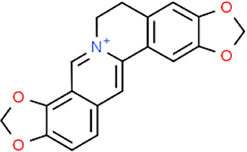 Coptisine	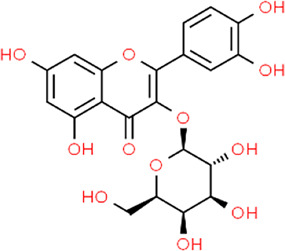 Hyperoside
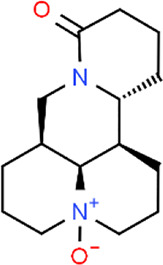 Oxymatrine	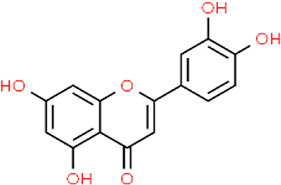 Luteolin	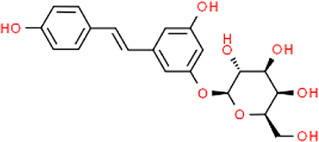 Polydatin	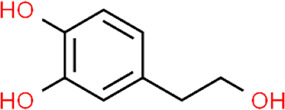 Hydroxytyrosol

## 2 Effects and Mechanisms of Natural Products on Apoptosis in UC

### 2.1 Death Receptor-Mediated Pathway

The apoptosis pathway activated by death receptors is also known as the “extrinsic pathway”. The ligand-bound death receptors refer to the proteins of tumor necrosis factor superfamily, such as tumor necrosis factor receptor, Fas, and tumor necrosis factor-related apoptosis initiating ligand-receptor (Vanamee and Faustman, 2018). These receptors can be activated by binding to their ligands, such as TNF-α, FasL, and TRAIL. The activated death receptors recruit Fas-associated death domain (FADD), the adaptor protein, which binds to the receptor and interacts with pro-caspase-8 to form a complex Death-inducing Signaling Complex (DISC), leading to the auto-cleavage and activation of caspase-8. Activated caspase-8 initiates the executioner caspase-3 to trigger the apoptotic cascades (Thorburn, 2004; Valmiki and Ramos, 2009). On the other hand, there is a cross-linking between the extrinsic pathway and intrinsic pathway (mitochondria-dependent apoptotic pathway), in which caspase-8 plays a critical role. Bid, a member of the bcl-2 family that regulates mitochondrial apoptosis, is cleaved by caspase-8 to activate the subsequent apoptotic events (Kantari and Walczak, 2011). Previous studies have already found that death receptor-mediated apoptosis is involved in the mucosal defect in UC (Yan et al., 2001; Yukawa et al., 2002; Fan et al., 2020).

**FIGURE 1:**
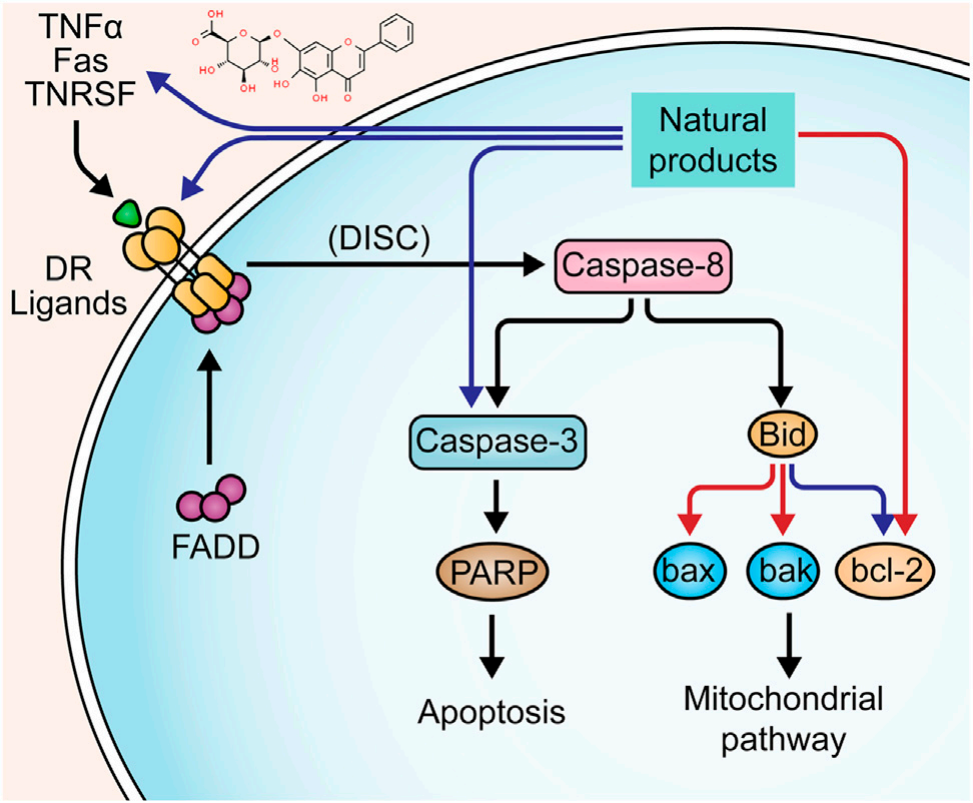
Natural products modulate apoptosis of IECs through death receptors mediated pathway.

In 2011, Liu et al. reported that *Sishen* Pill (2.5–10 g/kg), a prescription from traditional Chinese medicine (TCM), could inhibit epithelial apoptosis in rats through down-regulating Fas/FasL and up-regulating bcl-2 in colon tissues (Liu et al., 2011). In the same year, a study by Liu et al. found that Iridoid Glycosides (80–240 mg/kg), a fraction of *Folium syringae* [Myrtaceae: *Syringa vulgaris L.*] leaves, ameliorated epithelial apoptosis in experimental colitis of rats by modulating the expressions of Fas, FasL, caspase-3, bax, and bcl-2 (Liu and Wang, 2011). In 2016, a study by Yan et al. showed that a TCM prescription, *Wumei* Pill (13.3–53.2 g/kg), could inhibit the excessive apoptosis in colonic epithelial cells of rats via decreasing Fas, FasL, and caspase-3 (Shuguang. et al., 2016). Another study by Hui et al. also confirmed that *Wumei* Pill could decrease the bax expression and increase bcl-2 expression, exerting anti-apoptotic effects on colonic epithelial cells (Yi. et al., 2016). *In vitro* and *in vivo* studies on baicalin, a bioactive constituent from the root of *Scutellariae radix* [Lamiaceae: *Scutellaria baicalensis Georgi*], showed its anti-apoptotic activity, and the potential mechanisms were correlated to the down-regulation of Fas and FasL (Yao et al., 2016). The effects and mechanisms of natural products on death receptors-mediated apoptosis of UC are summarized in Figure 1.

### 2.2 Mitochondria-dependent Apoptotic Pathway

**FIGURE 2:**
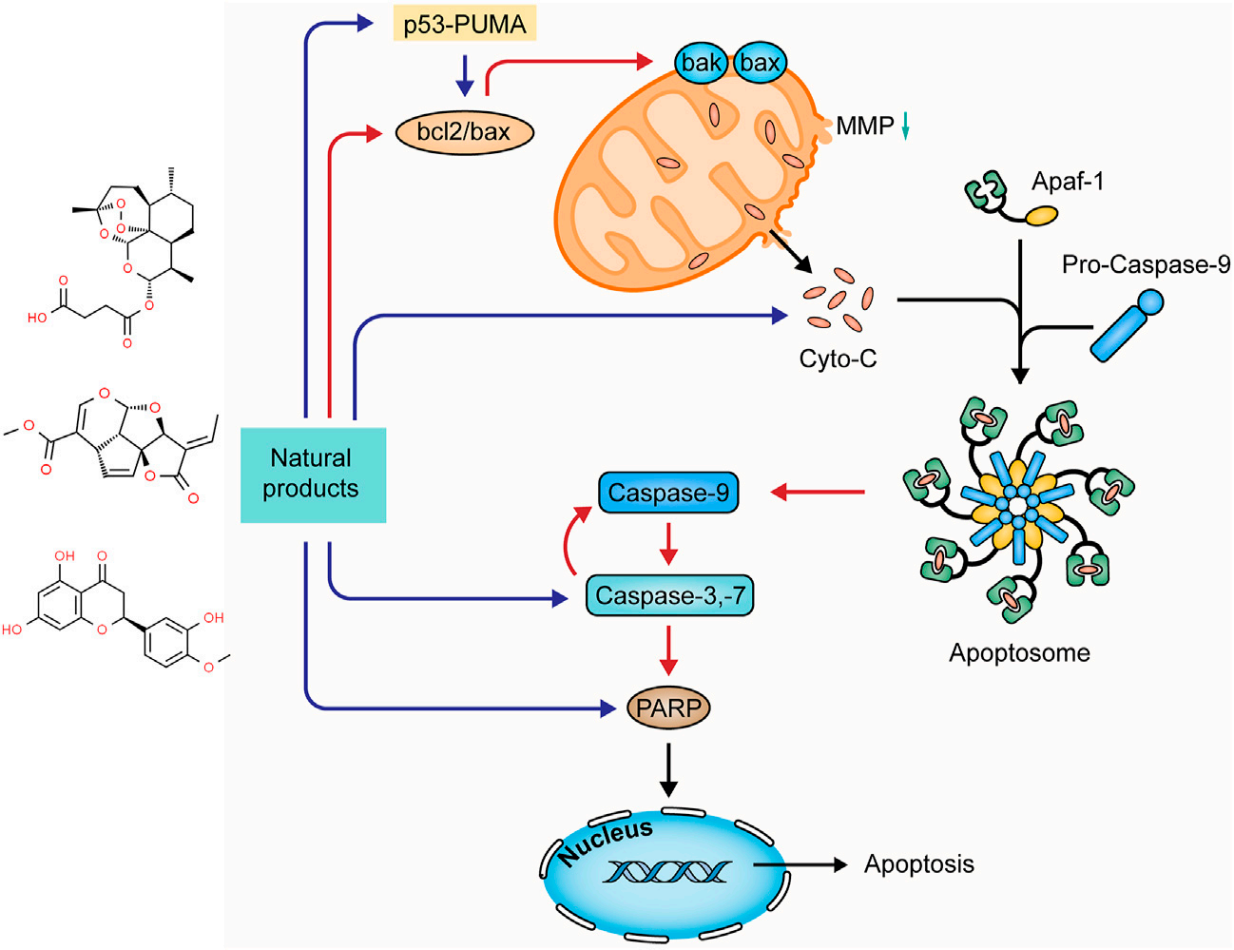
Natural products modulate apoptosis of IECs through mitochondria-dependent pathway.

Diverse apoptotic stimuli may evoke the decrease of mitochondrial membrane potential (MMP) and the release of pro-apoptotic proteins to activate the mitochondria-dependent apoptotic pathway (intrinsic pathway) (Thorburn, 2004; Elmore, 2007). The MMP integrity is regulated by the bcl-2 family composed of the pro-apoptotic members (e.g. bax, bad, bak, bim, and bid) and anti-apoptotic ones (e.g. bcl-2 and bcl-xL) (Jeong and Seol, 2008). Activating such apoptotic signaling pathways as p53-PUMA and death receptor pathways may enhance the pro-apoptotic proteins and reduce the anti-apoptotic proteins with a decrease in the MMP, disrupting the balance in the bcl-2 family. Increased mitochondrial membrane permeability induces the release of cytochrome-c (Cyt-c), which interacts with apoptosis protease-activating factor 1 (Apaf-1) to activate caspase-9 (Youle and Strasser, 2008; Qiu et al., 2011; Estaquier et al., 2012). Activated caspase-9 initiates pro-caspase-3 and -7, and in turn, the activated caspase-3 evokes pro-caspase-9, forming positive feedback. The activated executioner caspases cleave the downstream substrates, such as poly ADP-ribose polymerase (PARP), lamin, and fodrin, resulting in DNA fragmentation and apoptotic body formation (Fan et al., 2005). Increasing studies demonstrated that many natural products could mediate mitochondria-dependent apoptosis in UC. The effects and mechanisms of natural products on the mitochondria-dependent apoptotic pathway of UC are summarized in Figure 2.

#### 2.2.1 Extracts

In 2014, a study by Zhao et al. revealed that *Laggera Alata* [Asteraceae: *Laggera alata (D.Don) Sch. Bip. ex Oliv.*] Flavone (LAF) (100–400 mg/kg) could dramatically reduce apoptosis of colonic epithelial cells in trinitro-benzene-sulfonic acid (TNBS)-induced UC rats with the down-regulation of cyt-c, caspase-9, -3, and bcl-2/bax ratios (Xiaobin. and Xiaodong., 2014). In 2016, an investigation by Dong and Lu showed that Aucklandia [Asteraceae: *Dolomiaea costus (Falc.) Kasana and A.K.Pandey*] and Coptis [Ranunculaceae: *Coptis chinensis Franch.*] Pills (ACP) (1.6 g/kg) alleviated colonic epithelial apoptosis in TNBS-induced UC rats through decreasing bax expression and increasing bcl-2 expression (Yan. and Jingen., 2016). In 2018, Shi et al. found that a TCM clinical prescription, *Qingchang Wenzhong* granule (QCWZG), exerted anti-apoptotic effects (0.42–2.20 g/kg) in attenuating DSS-induced colitis rats via inhibiting bax and caspase-3 expressions and enhancing bcl-2 expression (Shi et al., 2019). In 2019, a study by Ma et al. indicated that Indigo [Brassicaceae: *Isatis tinctoria subsp. tinctoria*], one of the popular TCM botanical drugs, could reduce intestinal mucosa damage in experimental UC rats (200–800 mg/kg), and its mechanisms were associated with the down-regulation of caspase-3 and bax and the up-regulation of bcl-2 (Wenqiang et al., 2019). In 2020, an experiment by Hassanshahi et al. demonstrated that Aloe Vera Gel (AVG) could reduce cell apoptosis in the colon of acetic-acid-reduced colitis rats with a decreased bax and increased bcl-2 expressions (Hassanshahi et al., 2020). Yang et al. reported that the combination treatment with Coptidis Rhizoma [Ranunculaceae: *Coptis chinensis Franch.*] and Magnoliae Officinalis Cortex [Magnoliaceae: *Magnolia officinalis Rehder and E.H.Wilson*] (1, 2, and 4 g/kg) could protect colonic mucosa from apoptosis by decreasing bax and caspase-3 in TNBS-induced experimental rats (Xian-juan. et al., 2020). In 2021, Helal et al. elucidated the protective effects of graviola [Annonaceae: *Annona muricata L.*]. They observed that graviola treatment (100 mg/kg) attenuated apoptosis by modulating the expressions of bcl-2, bax, and caspase-3 (Helal and Abd Elhameed, 2021).

#### 2.2.2 Isolated Metabolites

Hesperetin is a flavonoid compound found in many citrus fruits. In 2019, an investigation by Polat and Karaboga suggested that hesperetin treatment (100 mg/kg) could improve the histopathological changes in the colon mucosa of TNBS-induced UC rats through down-regulating bax and caspase-3 (Polat and Karaboga, 2019). In the same year, a study by Pan et al. showed that Astragalus Polysaccharide (AP) (200 mg/kg), one of the main constituents in *Astragalus mongholicus* [Fabaceae: *Astragalus mongholicus Bunge*], could alleviate colonic epithelial defect by decreasing bax expression and increasing bcl-2 expression (Weijie et al., 2019). Artesunate (ARS) is a semisynthetic derivative of Artemisinin. A study by Yin et al. demonstrated that ARS (30 mg/kg) suppressed apoptosis in colon tissues of DSS-induced colitis rats and notably protected epithelial integrity via inhibiting bax and caspase-3 and enhancing bcl-2 (Yin et al., 2020). In 2021, Plumericin, a major bioactive constituent of *Himatanthus sucuuba* [Apocynaceae: *Himatanthus articulatus (Vahl) Woodson*], was reported by Rapa to exert anti-apoptotic effects and protect the intestinal epithelium and its barrier function *in vitro* (0.5–2 μg) and *in vivo* (3 mg/kg), and its potential mechanisms were correlated to the decrease of bax and caspase-3 and the increase of bcl-2 and bcl-xL (Rapa et al., 2021).

### 2.3 Endoplasmic Reticulum Stress Mediated Pathway

**FIGURE 3:**
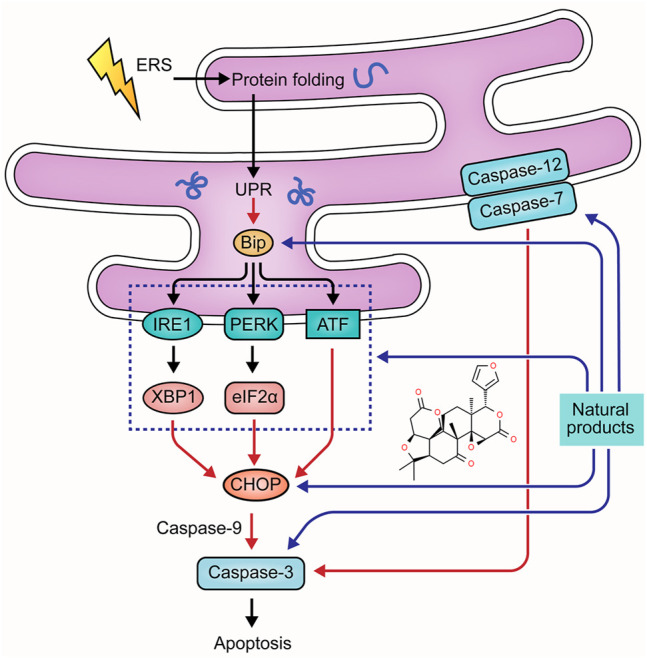
Natural products modulate apoptosis of IECs through ERS-mediated pathway.

Disrupted epithelial cell populations and functions can affect mucosal homeostasis of UC, leading to Endoplasmic reticulum stress (ERS). The protein-folding capacity of the endoplasmic reticulum is decreased, causing the unfolded protein response (UPR). This process can up-regulate the expression of chaperone proteins-encoding genes, such as glucose-2 regulated protein 78kD (GRP78) and Bip, triggering the downstream signaling of UPR, namely IRE1-XBP1, PERK-eIF2α, and ATF pathway. Activation of these pathways contributes to an increase in the C/EBP-homologous protein (CHOP), the bcl-2-interacting mediator of cell death (Bim), and the p53 up-regulated modulator of apoptosis (PUMA) to promote apoptosis (Hetz, 2012; Cao, 2016). ERS also induces apoptosis through the caspase-12 pathway. Pro-caspase-12 is pre-located on the cytoplasmic side of the ER, which can be cleaved in response to ERS. Caspase-12 can activate caspase-3, -9, and -7, directly inducing apoptosis (Tan et al., 2006; Liu et al., 2013). The role of ERS in UC pathogenesis has been recognized for decades, and numerous studies have yielded considerable evidence that natural products can protect intestinal epithelial cells from UC-induced apoptosis. The improvement effects and potential mechanisms of natural products on ERS-mediated apoptosis are summarized in Figure 3.

#### 2.3.1 Extracts

In 2021, Shen et al. found that Gancao Xiexin Decoction (10–40 μL) could inhibit the activation of PERK- elF2α-CHOP apoptotic signaling pathway to reduce the apoptosis of epithelial cells in UC, decreasing intestinal epithelial permeability and thus protecting intestinal mucosal barrier homeostasis (Yan et al., 2021).

#### 2.3.2 Isolated Metabolites

Berberine (BBR) is one of the bioactive components in *Coptis Chinensis* [Ranunculaceae: *Coptis chinensis Franch.*]. In 2018, Shen et al. reported that BBR treatment (100–200 mg/kg) could decrease apoptosis in intestinal epithelial cells of UC rats, indicating that the anti-apoptotic effect of BBR was associated with the down-regulation of caspase-12 and -3 (Yan. et al., 2018). In 2020, Yan et al. further explored the anti-apoptotic mechanisms of BBR, and their results suggested that this component (10 ml/kg) also down-regulated GRP78 to alleviate UPR (Yan et al., 2020). *In vitro* and *in vivo* investigation of Shen et al. demonstrated glycyrrhizin (0.5–2.0 mmol/L) could regulate ERS-evoked intestinal epithelial apoptosis and protect cells from apoptosis by decreasing GRP78, caspase-12, and caspase-3 (Yan and Bin., 2020). In 2021, a study reported Ginsenoside Rb1 (20 and 40 mg/kg), the major ginsenoside in ginseng [Araliaceae: *Panax ginseng C.A.Mey.*] with multiple pharmacological activities, significantly alleviated ERS in DSS-induced UC rats and TNBS-stimulated rat intestinal epithelial cells through decreasing GRP78, PERK, CHOP, caspase-12, and caspase-3 (Dong et al., 2021). Song et al. found that limonin, a tetracyclic triterpenoid compound obtained from plants of *Rutaceae* and *Meliaceae*, could improve colon pathology both *in vitro* and *in vivo* by inhibiting the PERK-ATF4-CHOP pathway to relieve ERS and subsequent cell apoptosis (Song et al., 2021). ARS has been demonstrated to alleviate UC by multiple pathways. Besides regulating mitochondria-dependent apoptosis, it also suppressed the activation of PERK-eIF2α-ATF4-CHOP and IRE1α-XBP1 signaling pathways to prevent ERS-mediated apoptosis in colon tissues (Yin et al., 2021).

### 2.4 MAPK-Mediated Apoptotic Pathway

**FIGURE 4:**
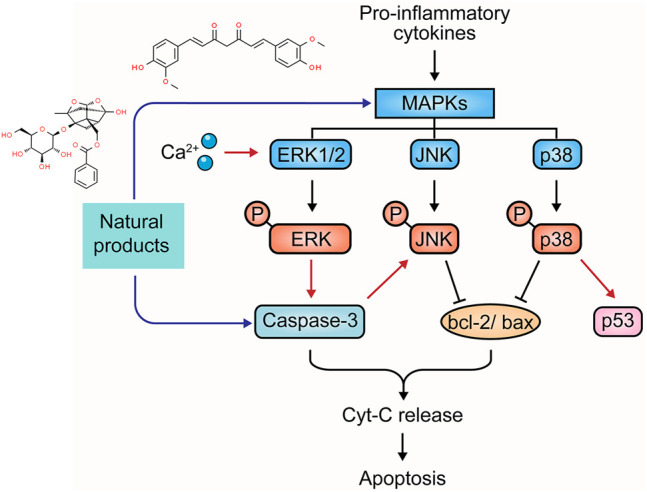
Natural products modulate apoptosis of IECs through MAPK mediated pathway.

Mitogen-activated protein kinase (MAPK) family members conventionally include extracellular-regulated kinase (ERK1/2), c-Jun N-terminal kinase (JNK), p38 MAPK, and ERK5 (Sun et al., 2015). They play pivotal roles in transduction extracellular stimuli into cellular responses of cell growth, migration, proliferation, differentiation, and apoptosis. ERK can be stimulated by growth factors and cytokines in UC pathology, leading to phosphorylation. The ERK signaling can play anti-apoptotic and pro-apoptotic roles depending on the stimuli (Li et al., 2014). JNK and p38 MAPK regulate several bcl-2 family proteins. One of the best-known transcription factors, p53, is also modulated by JNK/p38MAPK cascades to promote apoptosis. In addition, JNK/p38MAPK has been reported to be associated with the activation of caspase cascades (Yue and Lopez, 2020). Currently, MAPKs are considered a potential target in the treatment of UC. The potential effectiveness and mechanism of natural products on MAPK-mediated apoptosis are summarized in Figure 4.

#### 2.4.1 Extracts

Sishen Wan (SSW) is a notable TCM formula and has often been used in UC treatment. In 2013, Zhao et al. reported that SSW intervention (5 g/kg) in TNBS-induced colitis rats decreased colonic epithelial apoptosis by suppressing p38MAPK, p53, caspase-3, c-jun, c-fos, and bax expressions (Zhao et al., 2013). In 2016, a study by Taya et al. demonstrated that the extract (50 and 200 mg/kg) of Spirogyra neglecta, a freshwater green alga in the northern provinces of Thailand, diminished apoptosis of colonic epithelial cells in colitis mice via suppressing p38, ERK1/2, and MAP2K1 (Taya et al., 2016). Indirubin and Isatin are bioactive components in Qin Dai [Brassicaceae: *Isatis tinctoria subsp. tinctoria*]. In 2018, an investigation by Gao et al. demonstrated that the combination of Indirubin and Isatin inhibited cell apoptosis in DSS-induced UC mice through mediating the MAPK pathway, decreasing caspase-3, and increasing bcl-2 (Gao et al., 2018). *Qing Chang Hua Shi* granule (QCHS) also showed inhibitory effects on reducing UC-induced colonic apoptosis *in vitro* and *in vivo* by mediating MEK/ERK pathway and decreasing the expression of such apoptosis-related proteins as bax, bcl-2, caspase-3, -9, Fas, and Fas-L (Zhu et al., 2019a). In 2020, Sharma et al. reported that the extract of *Berberis lycium Royle* [Berberidaceae] fruit (125–500 mg/kg) could modulate intestinal epithelial cell apoptosis in mice through the inhibition of *p*-JNK and p-p38, increase of bcl-2, and decrease of bax, suggesting that it might be a viable candidate for UC treatment (Sharma et al., 2020).

#### 2.4.2 Isolated Metabolites

Curcumin is a major constituent of medicinal turmeric [Zingiberaceae: *Curcuma longa L.*]. A study in 2013 revealed that curcumin (100 mg/kg) could reduce colon injury in UC rats through the modulation of p38-and JNK-MAPK pathways (Topcu-Tarladacalisir et al., 2013). In 2015, Soubh et al. reported that Geraniol, a natural monoterpene alcohol (250 mg/kg), hindered apoptosis in TNBS-induced UC rats by suppressing p38 and caspase-3 expressions. Ger also up-regulated PPARγ, a transcriptional factor whose down-regulation is highly associated with the activation of the p38MAPK pathway (Soubh et al., 2015). Paeoniflorin (PA) is one of the major bioactive components in Paeony [Paeoniaceae: *Paeonia lactiflora Pall.*] root. In 2017, Gu et al. revealed that PA treatment (15–45 mg/kg) for experimental colitis mice could ameliorate the apoptosis in colitis tissues through inhibiting MAPK/NF-κB pathway (Gu et al., 2017). Moreover, a study by Li et al., in 2020 showed that PA down-regulated bax, caspase-3, and caspase-9 and up-regulated bcl-2 to protect UC-induced apoptosis (Lanzhen. et al., 2020). Chlorogenic acid is found in coffee and various TCM botanical drugs, such as honeysuckle [Caprifoliaceae: *Lonicera japonica Thunb.*], hawthorn [Rosaceae: *Crataegus pinnatifida Bunge*], eucommia [Eucommiaceae: *Eucommia ulmoides Oliv.*], and chrysanthemum [Asteraceae: *Chrysanthemum x morifolium (Ramat.) Hemsl*.]. Gao et al. reported that Chlorogenic acid (30–120 mg/kg) could significantly alleviate colonic tissue apoptosis and inflammation via the mediation of the MAPK/ERK/JNK signaling pathway (Gao et al., 2019). Anemoside B4, a bioactive triterpenoid saponin isolated from Chinese pulsatilla [Ranunculaceae: *Pulsatilla chinensis (Bunge) Regel*], was demonstrated to exert anti-apoptotic effects on UC through inhibiting p53, caspase-3, and bax expressions and the S100A9/MAPK/NF-κB signaling pathway (Yong. et al., 2020; Zhang et al., 2021).

### 2.5 NF-κB Mediated Apoptotic Pathway

**FIGURE 5:**
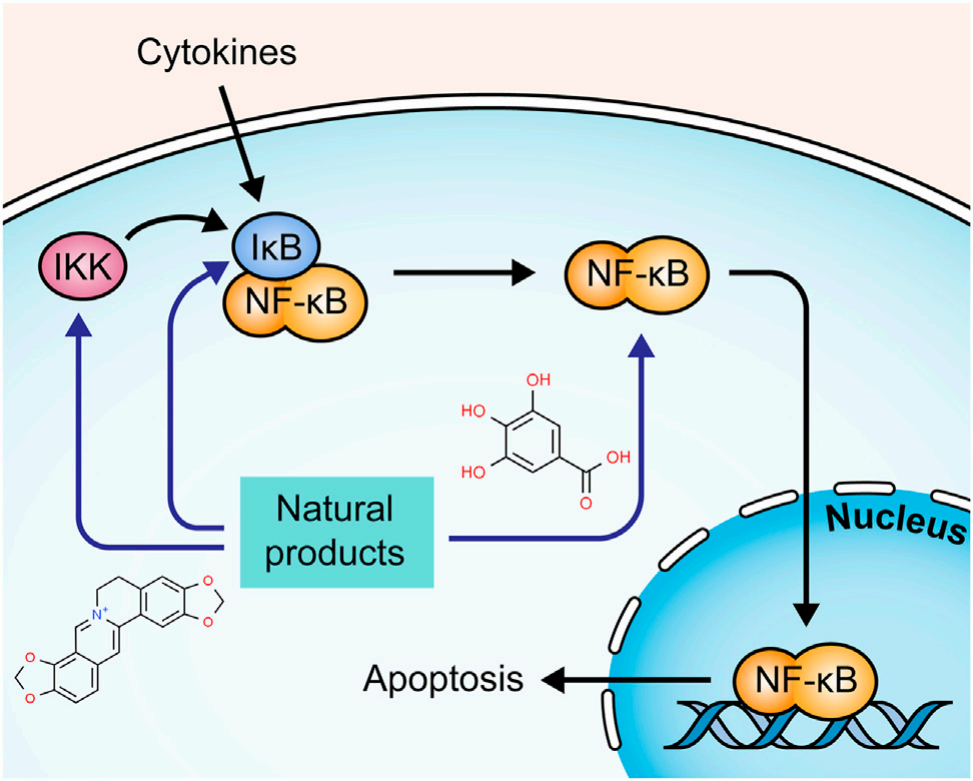
Natural products modulate apoptosis of IECs through NFκB mediated pathway.

The transcription factor nuclear factor-kappaB (NF-κB) is also involved in the regulation of cell death. Under resting conditions, NF-κB is sequestered in the cytoplasm through interaction with IκB, an inhibitory protein. In the presence of NF-κB-activating stimuli, such as proinflammatory cytokines, IκB can be phosphorylated by IκB kinase (IKK) and degraded, leading to the translocation of NF-κB to the nucleus (Heyninck and Beyaert, 2001; Aranha et al., 2007). Activated NF-κB contributes to the transcription of multiple genes to regulate apoptosis induced by extrinsic and intrinsic apoptotic pathways (Baldwin, 2012; Liu et al., 2012). The potential mechanisms of natural products on NF-κB mediated apoptosis are summarized in Figure 5.

#### 2.5.1 Extracts

A study by Liu and Wang showed that the Iridoid Glycosides fraction inhibited IkBα phosphorylation and IKK activity in intestinal epithelial cells, regulating the NF-κB signaling pathway (Liu and Wang, 2011). Another study by Zhang et al. presented similar results of IG in regulating the NF-κB signaling pathway (Zhang Y. et al., 2020). Corilagin is a major Gallotannin found in many medicinal plants. In 2013, a study by Xiao et al. demonstrated that Corilagin (7.5–30 mg/kg) suppressed the degradation of IκBα and down-regulated caspase-3 and -9, reducing apoptosis in colon tissues of UC mice (Xiao et al., 2013). Portulaca [Portulacaceae: *Portulaca oleracea L.*] is a wildly used botanical drug in TCM. In 2018, an investigation by Kong et al. revealed that Portulaca extract could alleviate colitis in mice and mediate colonic cell apoptosis through inhibiting the NF-κB pathway with decreased expressions of bax and caspase-3 and increased expression of bcl-2 (Kong et al., 2018). A study by Lin et al. displayed that QingBai decoction (QBD), a TCM prescription, effectively reduced apoptosis in the colon of DSS-induced UC mice via regulating the NF-κB pathway and decreasing caspase-3 (Lin et al., 2019). Gallic acid is widely present in many plants and fruits. In 2019, Zhu et al. found that it inhibited UC-induced apoptosis *in vitro* and *in vivo* by suppressing the expressions of *p*-IκBα and p-NF-κB, decreasing caspase-3 and -9, and increasing bcl-2 (Zhu et al., 2019b). Chickpea [Fabaceae: *Cicer arietinum L.*] is a staple food crop in tropical and subtropical areas. In 2020, Kim et al. reported that its ethanol extract (100 and 200 mg/kg) exerted a protective effect on DSS-induced apoptosis in colon tissue by the inhibition of nuclear factor-kappa B (NF-κB) and signal transducer and activator of transcription 3 (STAT3) (Kim et al., 2020). Canna [Cannaceae: *Canna x generalis L.H. Bailey*] is wildly used in folk medicine for the treatment of many diseases. In 2021, a study by Mahmoud et al. showed that its rhizome ethanol extract (100, 200 mg/kg) down-regulated NF-κB and caspase-3 expressions in colon tissues of colitis mice (Mahmoud et al., 2021).

#### 2.5.2 Isolated Metabolites

In 2010, Gu et al. demonstrated that Deoxyschisandrin (5 μg/ml), one of the lignan components of *Schisandra Chinensis* [Schisandraceae: *Schisandra chinensis (Turcz.) Baill.*] fruits, could inhibit apoptosis of intestinal epithelial cells, and the potential mechanisms were associated with the inhibition of IκB degradation and the subsequent NF-κB activation (Gu et al., 2010). A study by Shen et al. discovered that Baicalin (30–90 mg/kg) presented a significant anti-apoptotic effect on TNBS-induced UC rats and LPS-induced RAW264.7 cells through regulating the IKK/IKB/NF-kB signaling and the expressions of such apoptosis-related proteins as cyt-c, caspase-3, -9, bcl-2, and bax (Shen et al., 2019). In 2020, Motawea et al. showed that Oleuropein (350 mg/mg), a major component of *Olea europaea L*.[Oleaceae], reduced apoptosis in colon tissues of experimental UC rats via down-regulating the expression of NF-kB and bax and up-regulating bcl-2 (Motawea et al., 2020). In 2021, a study by Li et al. revealed that 6,7-Dihydroxy-2,4-Dimethoxyphenanthrene (CYP4, 60–240 mg/kg) from Chinese Yam [Dioscoreaceae: *Dioscorea oppositifolia L*.] could protect intestinal mucosa from apoptosis in DSS-induced colitis mice by suppressing NF-κB and caspase-3 expressions (Li et al., 2021). In the same year, Wang et al. stated that Coptisine (100 mg/kg), a major bioactive component from Rhizoma Coptidis [Ranunculaceae: *Coptis chinensis Franch.*], markedly alleviated DSS-induced apoptosis in intestinal epithelial cells of rats by restraining IκBα phosphorylation and NF-κB translocation, down-regulating bax and caspase-3, and up-regulating bcl-2 (Wang et al., 2021). Yu et al. observed that hyperoside (25–100 mg/kg), a flavonol glycoside isolated from plants of Hypericum and Crataegus, could inhibit TNBS-induced intestinal epithelial apoptosis in rats via decreasing NF-κB and caspase-3 (Yu et al., 2021). Yu and Qian reported that Deoxyschizandrin treatment (20–80 mg/kg) could reduce apoptosis of colonic epithelial cells in UC model mice, which might be attributed to the inhibition of TLR4/NF-κB signaling pathway and the regulation of bcl-2, bax, and caspase-3 (Yu and Qian, 2021).

### 2.6 P13K/Akt Pathway

**FIGURE 6:**
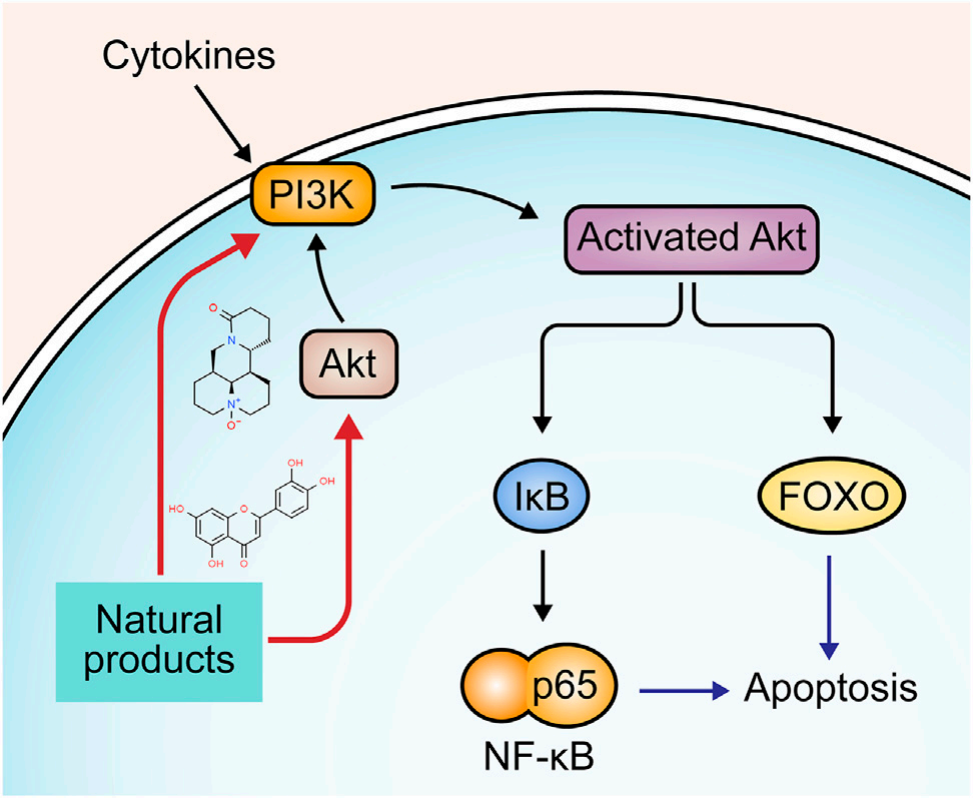
Natural products modulate apoptosis of IECs through P13K/Akt pathway.

Phosphatidylinositol 3-kinase (PI3K)/Akt signaling is implicated in multiple cellular processes, such as survival, proliferation, differentiation, and apoptosis (Vivanco and Sawyers, 2002). P13K can be activated by various cytokines and be recruited to the membrane. Akt, the downstream target protein of P13K, migrates to the membrane and activates the sequential phosphorylation of P13K. Activated Akt releases from the membrane to cytosol to phosphorylate fork-head transcription factor (FOXO), triggering the downstream signaling pathways that regulate many apoptotic genes related to the intrinsic and extrinsic pathway (Franke et al., 2003; Fresno Vara et al., 2004; Zhang et al., 2011). P13K/Akt can also activate NF-κB through phosphorylating IκB (Chen et al., 2017). The involvement of the P13K/Akt signaling pathway in UC pathogenesis has been well-documented (Huang et al., 2011). Recently, compelling evidence has revealed that several natural products alleviate apoptosis in UC through regulating P13K/Akt signaling pathway. The potential mechanisms of natural products on P13K/Akt mediated apoptosis are summarized in Figure 6.

In 2017, Chen et al. reported that Oxymatrine (25–100 mg/kg), an alkaloid derived from the root of the Sophora flavescens [Fabaceae: *Sophora flavescens Aiton*], could alleviate apoptosis through P13K/Akt pathway and exhibit potential therapeutic effects against DSS-induced colitis (Chen et al., 2017). In the same year, another study by Zhu et al. found that baicalin (20–100 mg/kg) reduced the expression of caspase-9 and FasL by regulating the P13K/Akt pathway (Zhu. et al., 2017). In 2018, an investigation by Wang et al. demonstrated that granules extracted from Costus root [Asteraceae: *Dolomiaea costus (Falc.) Kasana and A.K.Pandey*] (1000 mg/kg) could inhibit apoptosis of intestinal epithelial cells in UC rats via promoting the activities of P13K and Akt and subsequently down-regulating caspase-3 and bad while up-regulating bcl-2 and p53 (Wang et al., 2018). Luteolin is a common flavonoid in several plants, such as lemon, apple, and onion. In 2020, Vukelic et al. showed that luteolin (50–100 mg/kg) decreased caspase-3, -9, and PARP in UC mice, which may be related to Akt regulation (Vukelic et al., 2020).

### 2.7 Other Reported Pathways

Besides the major apoptotic pathways mentioned above, other mechanisms are also attributable to the anti-apoptotic activities of natural products on UC. For example, Aloe polysaccharide (AP) extracted from aloe vera [Asphodelaceae: *Aloe vera (L.) Burm. f.*] could effectively reduce the apoptosis in colonic tissues via inhibiting the JAK2/STAT-3 signaling pathway *in vivo* and *vitro* (Lin et al., 2017). Another study conducted in 2019 by Bu et al. revealed that Tripterygium glycosides (27 mg/kg) could attenuate intestinal mucosal apoptosis in UC rats through JAK2/STAT3 signaling pathway (Nan et al., 2019). Moreover, an investigation by Chen et al. demonstrated that *Chushi Jianpi* Decoction, a TCM prescription, could reduce intestinal epithelial apoptosis in colitis mice through inhibiting IL-10/JAK1/STAT3 pathway (Chen et al., 2021). Polydatin (15–45 mg/kg), a main component in Polygonum cuspidatum [Polygonaceae: *Reynoutria japonica Houtt.*], was reported to exert protective effects on DSS-induced apoptosis in mice by up-regulating the Sonic hedgehog signaling pathway, decreasing caspase-3 and bax, and increasing bcl-2 (Lv et al., 2018). In 2020, Yang et al. demonstrated that crocin (0.05–0.1 g/kg), a carotenoid compound derived from *Crocus sativus L.*(Iridaceae), could decrease bax and caspase3 and increase bcl-2 in the intestinal tissue of UC rats, and its potential mechanisms might be correlated with the down-regulation of the TLR4/MyD88 signaling pathway (Yang et al., 2020). In 2021, a study by Miao et al. revealed that Walnut oil (2.5 mg/kg) down-regulated the related gene proteins expression of the NLRP3/ASC/Caspase-1 pathway to inhibit apoptosis in DSS-induced colitis mice (Miao et al., 2021).

In addition, some natural agents have exerted therapeutic effects on UC-elicited apoptosis, but their mechanisms need to be further verified. In 2010, Pan et al. reported that polysaccharides of Portulaca oleracea (200mg/0.33 ml) could reduce intestinal epithelial apoptosis in TNBS-induced UC rats with decreased caspase-3 and -8 expression in the epithelium (Feng. et al., 2010). Interestingly, feeding DSS-induced colitis rats with honey (5 g/kg) could improve colonic histology by decreasing caspase-3 in colons (Nooh and Nour-Eldien, 2016). In 2021, Elmaksoud et al. demonstrated that Hydroxytyrosol (50 mg/kg), one of the main alcoholic compounds of the olive leaves extract, down-regulated the expression of bax and up-regulated that of bcl-2 in the colons of acetic acid-induced colitis rats (Elmaksoud et al., 2021). Tanshinol, a bioactive ingredient in DanShen [Lamiaceae: *Salvia miltiorrhiza Bunge*], was reported by Zhu et al. to alleviate apoptosis in UC model cells through promoting very low-density lipoprotein receptor expression (Zhu et al., 2021).

## 3 Conclusions and Perspectives

Natural products refer to a wide range of bioactive extracts or isolated metabolites from natural materials. Their bioactivities are currently of great interest in many research fields (Ekiert and Szopa, 2020) and may yield promising pharmacological approaches for the prevention and treatment of UC due to their multiple regulatory effects with few adverse effects (Nascimento et al., 2020). In recent years, converging lines of evidence have demonstrated that apoptosis of IECs is highly associated with the occurrence and development of UC. This review reported that multiple natural products have anti-apoptotic activities *in vitro* and *in vivo* to protect intestinal epithelial cells against apoptosis in UC. Furthermore, their potential mechanisms are closely associated with the regulation of multiple apoptosis-related signaling pathways, including death-receptor mediated pathway, mitochondrial-dependent pathway, ERS-mediated pathway, MAPK-mediated pathway, NF-κB mediated pathway, P13k/Akt pathway, and other reported pathways such as JAK/STAT3 and NLRP3/ASC/Caspase-1. Thus, it is rational to presume that natural products may yield promising therapeutic agents to treat UC patients by modulating apoptosis of IECs.

Although many natural products have been demonstrated to be the potential candidates for UC treatment by targeting intestinal epithelial apoptosis, more sophisticated works in preclinical and clinical investigations need to be performed to research and develop effective pharmacotherapies. First, studies on the pharmacokinetics and pharmacodynamics of these natural agents are insufficient. Recently, with the increasing attention drawn on natural products, great attention has been paid to metabolism and pharmacokinetics research (Zeng et al., 2017), laying a foundation for subsequent research of toxicology and medication safety. Second, systematic evaluation for the toxicity and safety of natural products remains scarce. Though these natural plants and botanical drugs have been wildly used for thousands of years, well-designed studies for critical evaluation of safety are imperative for developing novel and effective pharmacotherapeutic agents. More concerns need to be paid on the potential toxicity and adverse effects of natural products (Wu et al., 2016; Byard et al., 2017; Liu et al., 2020). Third, current research of natural products targeting apoptosis in UC has primarily focused on their *in vivo* and *in vitro* effects and mechanisms. Well-designed clinical trials with high methodological quality are urgently needed for further verification of these natural products. Lastly, the exploration of mechanisms of some natural products is still in the preliminary stage, and the specific targets and signaling pathways require further elucidation. In addition, some of the above-mentioned natural products, such as baicalin, Indirubin, and Paeoniflorin, are reported to modulate apoptosis through multiple pathways, and their underlying interactions or crosstalk with the core-target network of UC are worthy of further exploration.

In summary, we expect that this review will provide helpful information to understand the effects of natural products and their pharmacological mechanisms in regulating intestinal epithelial apoptosis of UC. These natural extracts and isolated metabolites are of potential value in clinical UC management. We also expect more researchers and clinicians to pay close attention to this field and conduct more relevant studies and trials.
